# Systems biology of acidophile biofilms for efficient metal extraction

**DOI:** 10.1038/s41597-020-0519-2

**Published:** 2020-07-07

**Authors:** Antoine Buetti-Dinh, Malte Herold, Stephan Christel, Mohamed El Hajjami, Sören Bellenberg, Olga Ilie, Paul Wilmes, Ansgar Poetsch, Wolfgang Sand, Mario Vera, Igor V. Pivkin, Mark Dopson

**Affiliations:** 1 Institute of Computational Science, Faculty of Informatics, Università della Svizzera italiana, Lugano, Switzerland; 2 Swiss Institute of Bioinformatics, Lausanne, Switzerland; 3 Department of Chemistry and Biomedical Sciences, Linnaeus University, Kalmar, Sweden; 4 Centre of Excellence for Biomaterials Chemistry, Linnaeus University, Kalmar, Sweden; 5 Centre for Ecology and Evolution in Microbial Model Systems, Linnaeus University, Kalmar, Sweden; 6 Luxembourg Centre for Systems Biomedicine, University of Luxembourg, Belvaux, Luxembourg; 7 Plant Biochemistry, Ruhr University Bochum, Bochum, Germany; 8 Center for Marine and Molecular Biotechnology, QNLM, Qingdao, China; 9 College of Marine Life Sciences, Ocean University of China, Qingdao, China; 10 Faculty of Chemistry, Biofilm Centre, University Duisburg-Essen, Essen, Germany; 11 College of Environmental Science and Engineering, Donghua University, Shanghai, People’s Republic of China; 12 Mining Academy and Technical University Freiberg, Freiberg, Germany; 13 Institute for Biological and Medical Engineering. Schools of Engineering, Medicine & Biological Sciences, Pontificia Universidad Católica de Chile, Santiago, Chile; 14 Department of Hydraulic & Environmental Engineering, Pontificia Universidad Católica de Chile, Santiago, Chile

**Keywords:** Soil microbiology, Environmental impact

## Abstract

Society’s demand for metals is ever increasing while stocks of high-grade minerals are being depleted. Biomining, for example of chalcopyrite for copper recovery, is a more sustainable biotechnological process that exploits the capacity of acidophilic microbes to catalyze solid metal sulfide dissolution to soluble metal sulfates. A key early stage in biomining is cell attachment and biofilm formation on the mineral surface that results in elevated mineral oxidation rates. Industrial biomining of chalcopyrite is typically carried out in large scale heaps that suffer from the downsides of slow and poor metal recoveries. In an effort to mitigate these drawbacks, this study investigated planktonic and biofilm cells of acidophilic (optimal growth pH < 3) biomining bacteria. RNA and proteins were extracted, and high throughput “omics” performed from a total of 80 biomining experiments. In addition, micrographs of biofilm formation on the chalcopyrite mineral surface over time were generated from eight separate experiments. The dataset generated in this project will be of great use to microbiologists, biotechnologists, and industrial researchers.

## Abstract (summary)

**Table:** 

Measurement(s)	biofilm formation • Proteome • protein expression data • RNA • transcriptome
Technology Type(s)	fluorescence microscopy • mass spectrometry • RNA sequencing
Sample Characteristic - Organism	Leptospirillum ferriphilum • Sulfobacillus thermosulfidooxidans • Acidithiobacillus caldus
Sample Characteristic - Environment	mine

Machine-accessible metadata file describing the reported data: 10.6084/m9.figshare.12173637

## Background & Summary

Biomining is an industrial biotechnology involving sulfide mineral dissolution for the recovery of metals such as copper, nickel, and gold^1^. For low-grade ores, biomining is often carried out in very large heaps whereby the ore is stacked on an impermeable membrane, acid and in some cases microbes are added to the surface, and solubilized metals are collected at the base of the heap that are subsequently recovered^2^. During biomining, the sulfide mineral is attacked by ferric ions that results in soluble metal ions and the resulting ferrous iron is re-oxidized by acidophilic microorganisms^3^ to complete the abiotic-biotic catalytic cycle^4^. Further products of the ferric iron attack on the metal sulfide are inorganic sulfur compounds that are oxidized to generate the acidity required by the acidophiles^5^.

One of the major challenges for further exploitation of industrial biomining is for copper recovery from the refractory mineral chalcopyrite (CuFeS_2_), the largest reserve of copper containing mineral in the world. The problems associated with chalcopyrite bioleaching (*i*.*e*., when the target metal forms part of the mineral matrix) are slow rates and poor total recoveries often attributed to passivation of the mineral surface that has recently been suggested to be by iron-oxyhydroxides^6^. One method to avoid chalcopyrite passivation is to carry out the bioleaching at low redox potentials and high temperatures^7,8^ and several methods have been suggested to maintain the redox potential in the desired range including controlling the oxygen concentration^9^ or utilizing a microbial community that maintains the potential in the desired range^10,11^. An additional critical factor for chalcopyrite bioleaching, especially in early stages of bioheap inoculation, is the attachment and biofilm formation on the mineral surface^12^. However, how to control the redox potential to achieve high copper dissolution rates and recoveries has not been solved in several square kilometer large industrial bioheaps.

**Fig. 1:**
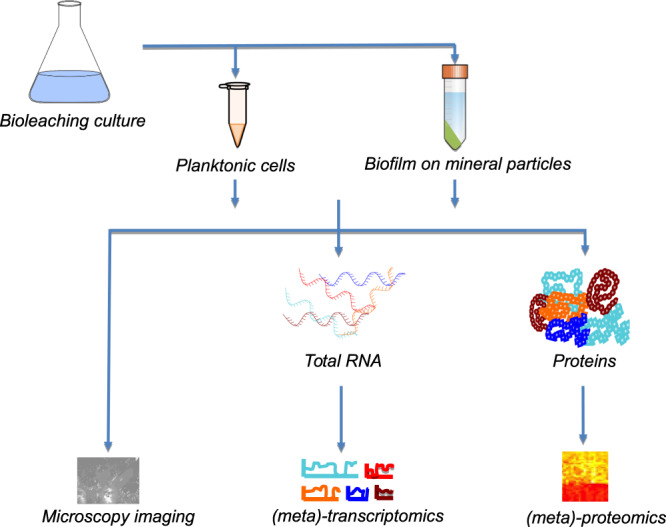
Workflow depicting the cultivation of (mixed) bioleaching bacteria cultures, proteins, and transcripts extraction and generation of microscopy, (meta)-transcriptomics, and (meta)-proteomics datasets.

In this Data Descriptor, we present data for (meta)-transcriptomes, (meta)-proteomes, microscope images, and the accompanying metadata of axenic and defined consortia of bioleaching bacteria growing in continuous cultures and in the presence of chalcopyrite mineral (Fig. 1). The data set contains twelve continuous culture samples (Table 1), omics data from 80 bioleaching experiments (Table 1), and microscopy images tracking biofilm formation on chalcopyrite grains for eight distinct conditions over time (Table 1). The complete data in this descriptor have not been previously reported although parts have been included in published articles. These include RNA transcripts and protein concentrations of *Leptospirillum ferriphilum*^T^ in axenic culture^13^, RNA transcripts and protein concentrations of simple defined mixed cultures^11^, microscopy images^14,15^, and on reverse engineering of omics data to generate gene regulatory networks^16^.

**Table 1:** Number of samples for which RNAseq and proteomics data are provided.

Condition	Combination of strains*	RNA samples	Protein samples	Both	Imaging
Cn	AXX	6	3	3	
Cn	LXX	3	0	0	
Cn	SXX	3	0	0	
M	AXX	2	0	0	yes
M	LXX	2	4	2	yes
M	S–AL**	2	4	1	yes
M	S–LX**	2	2	0	yes
P	ALX	3	0	0	
P	ASL	3	0	0	
P	ASX	11	20	11	yes
P	AXX	4	2	2	
P	LSX	3	4	3	yes
P	LXX	2	5	2	yes
P	S–AL**	3	5	3	yes
P	S–AX**	4	11	3	yes
P	SLX	4	0	0	yes
P	SXX	4	4	3	yes

Condition or cellular fraction indicates if the samples were derived from continuous culture (Cn) or bioleaching cultures and here either from the biofilm fraction (M) or the planktonic fraction (P). The number of samples for which RNA and proteins derived data could be generated are given in column 4 (Both).

*The combination of strains were composed of *A*. *caldus* (A), *L*. *ferriphilum* (L) and *S*. *thermosulfidooxidans* (S) and used as pure or mixed cultures, resulting in the following categories: A, L, S, AS, LS, and ASL (X stands for no species). A description corresponding to the strain composition and sample naming is found in the FAIRDOMHub repository (“SysMetEx – Dataset collection”^55^, “omics samples”).

**The dash sign (“–”) in the combination of strain names indicates sequential inoculation for pre-colonization by the species corresponding to the letter before the dash sign.

## Methods

These methods are expanded versions of descriptions in our related work^11–16^.

### Transcriptomics and proteomics

#### Mineral preparation for transcriptomics and proteomics

Chalcopyrite mineral concentrate from the Aitik copper mine (N 67°4′ 24″, E 20°57′ 51″) was provided by Boliden AB (Sweden). It was of high purity (>98%) and high copper content (29.5%) as revealed by aqua regia digestion and elemental analysis. The concentrate was sieved to a size fraction of 50–100 *μ*m and washed in three volumes of 0.1 M EDTA in 0.4 M NaOH for 10 min while stirring to remove iron and copper compounds resulting from mineral weathering^17,18^. After elemental sulfur was removed from the mineral surfaces by three washing iterations with one volume of acetone, the mineral was dried at 60 °C overnight, and subsequently autoclaved at 120 °C for 10 h under a nitrogen atmosphere to prevent changes in its structure. This procedure likely prevented the growth of contaminating microbes as flotation chemicals are toxic to the acidophile, *Sulfolobus metallicus*^19^.

#### Microbial species cultivation for transcriptomics and proteomics

Three bacterial acidophile species were used: *Leptospirillum ferriphilum*^T^ DSM 14647^20^, *Sulfobacillus thermosulfidooxidans*^T^ DSM 9293^21^, and *Acidithiobacillus caldus*^T^ DSM 8584^22^. Cells were maintained in the exponential growth phase at 38 °C in three separate axenic continuous cultures^11^ until inoculation for further experiments. Continuous cultures (1 L working volume) containing Mackintosh basal salt (MAC) medium^23^ and ferrous sulfate (100 mM) as electron donor were adjusted to pH 1.4 for *L*. *ferriphilum*, or with 5 mM potassium tetrathionate adjusted to pH 2.3 and pH 2.0 for *S*. *thermosulfidooxidans* and *A*. *caldus*, respectively. The continuous culture vessels containing MAC medium plus tubing, connectors *etc*. were autoclaved while the ferrous sulfate and potassium tetrathionate were sterile filtered (0.2 *μ*m pore size, cellulose acetate filter, PALL).

Bioleaching experiments were carried out and analyzed as previously reported^11^. Quadruplets of 100 mL MAC medium were adjusted to pH 1.8 by addition of sulfuric acid and supplemented with 2% (wt/vol) chalcopyrite concentrate. Different combinations of the three bacteria were inoculated in aliquots of 10^7^ cells per mL per species that were captured by 20 min centrifugation from the continuous cultures at 12,500 g. Sample inoculation occurred with all species simultaneously according to the three-letter sample names, in which the initial letters of the bacterial species was used (Tables 1 and 2), if not otherwise indicated. When the inoculation order was sequential, a dash sign (“–”) in the sample names indicates 48 h pre-colonization of the species corresponding to the first letter. For example, sample “ASL” indicates inoculation of the three species at the same time, while in sample “S–AL” *S*. *thermosulfidooxidans* was used as pre-colonizer and 48 h later *A*. *caldus* and *L*. *ferriphilum* were added.

**Table 2:** Identification rate of MS/MS spectra for the different samples.

Condition	Combination of strains*	MS/MS submitted	MS/MS Identified (%)
Cn	AXX	185932	24.63
M	LXX	166215	14.14
M	S–AL**	263940	20.61
M	S–LX**	78849	30.16
P	ASX	530117	16.78
P	AXX	152847	14.91
P	LSX	347396	29.66
P	LXX	229079	20.01
P	S–AL**	168878	25.32
P	SXX	161726	24.63
**Average**			**22**.**09**

*The combination of strains were composed of *A*. *caldus* (A), *L*. *ferriphilum* (L) and *S*. *thermosulfidooxidans* (S) and used as pure or mixed cultures, resulting in the following categories: A, L, S, AS, LS, and ASL (X stands for no species). A description corresponding to the conditions is found in the FAIRDOMHub repository (“SysMetEx – Dataset collection”^55^, “Sample Identification Code”).

**The dash sign (“–”) in the combination of strain names indicates sequential inoculation for pre-colonization by the species corresponding to the letter before the dash sign.

Cell counts were obtained using a Neubauer improved counting chamber. Experiments using single, binary, and tertiary combinations of the microbial species along with a sterile control were carried out in triplicates. Cultures were incubated at 38 ± 2 °C with slow shaking at 120 rpm. Bioleaching experiments were monitored during a period of 14–20 days by measuring the redox potential with respect to an Ag/AgCl electrode. At the termination of the experiment, cell mass for protein and RNA extraction were obtained.

#### RNA and protein extraction

To ensure no degradation of biomolecules occurred, the bioleaching vessels were allowed to settle for 5 min, before the supernatant (75 mL) was removed and immediately mixed with 75 mL ice cold sterile MAC medium. The resulting mixture was then centrifuged (20 min, 12,500 g, and 4 °C). The cell pellets were washed twice by re-suspending in sterile, ice-cold MAC medium, flash frozen in liquid nitrogen, and used for biomolecular extractions according to Roume *et al*.^24^ with the alteration that metabolites were not extracted. RNA samples were sequenced at Science for Life Laboratory (Stockholm, Sweden) while the precipitated protein fraction was analyzed by mass spectrometry.

#### RNA sequencing and transcript analysis

The Illumina TruSeq Stranded mRNA kit was used for rRNA depletion and library preparation. RNA sequencing reads with an average length of 126 bases were obtained by Illumina HiSeq2500. Raw reads were filtered with Trimmomatic v0.32^25^ and aligned to a concatenation of the three reference genomes (*A*. *caldus* DSM8584: GCF_000175575.2; *S*. *thermosulfidooxidans* DSM 9293: GCF_900176145.1; *L*. *ferriphilum* DSM 14647: GCF_900198525.1) using Bowtie-2 v2.3.2^26^. Sequencing reads that mapped to protein coding sequences were quantified with FeatureCounts of the subread package v1.5.1^27^ and expressed as transcripts per million (TPM) for the *A*. *caldus*, *S*. *thermosulfidooxidans*, and *L*. *ferriphilum* genomes, respectively. Similarly, to compare samples of different compositions, read counts were normalized per reference genome using DESeq 2 v1.16.1^28^ and compared accordingly to obtain log_2_-fold changes (Log_2_FC)^29^.

#### Proteomics and protein identification

A total of five protein extracts from the continuous culture and batch experiments were precipitated in acetone, dried, and dissolved in 20 *μ*L of 6 M urea - 2 M thiourea by vortexing. Cysteines reduction was achieved by 30 min incubation at room temperature with 1 *μ*L of 1 M dithiothreitol, followed by 20 min alkylation with 1 *μ*L of 550 mM iodoacetamide in the dark. Proteins were digested at room temperature for 3 h with lysyl endopeptidase (Wako) at a protease/protein ratio of 1:100, thereupon urea was diluted to 2 M with 50 mM ammonium bicarbonate and digestion continued with sequencing grade trypsin (Promega) for 12 h at room temperature at a protease/protein ratio of 1:100. The resulting peptides were extracted from the gel with acetonitrile and stored on stop-and-go extraction (STAGE) tips prior to mass spectrometry^30^.

Mass spectra were recorded with Xcalibur software 3.1.66.10 (Thermo Scientific). The continuous culture samples were analyzed with an EASY-nLC 1000 liquid chromatography (LC) system (Thermo Scientific) and a Q-Exactive HF mass spectrometer (Thermo Scientific), as described previously^31^. Mineral culture samples were analyzed by using a nanoACQUITY gradient ultraperformance liquid chromatography (UPLC) pump system (Waters, Milford, MA, USA) coupled to an LTQ Orbitrap Elite mass spectrometer (Thermo Fisher Scientific Inc., Waltham, MA, USA). An UPLC HSS T3 M-class column (1.8 *μ*m, 75 *μ*m by 150 mm; Waters, Milford, MA, USA) and an UPLC Symmetry C 18 trapping column (5 *μ*m, 180 *μ*m by 20 mm; Waters, Milford, MA, USA) were used for LC in combination with a PicoTip emitter (SilicaTip, 10 *μ*m internal diameter [i.d.]; New Objective, Woburn, MA, USA). To elute the peptides, a linear gradient was employed with increasing concentrations of buffer B (0.1% formic acid in acetonitrile [ULC/MS grade]; Biosolve, Netherlands) from 1% to 95% within 166.5 min, followed by a linear gradient from 1% acetonitrile within 13.5 min (1% buffer B from 0 to 10 min, 5% buffer B from 10 to 161 min, 40% buffer B from 161 to 161.5 min, 85% buffer B from 161.5 to 166.5 min, 95% buffer B from 166.5 to 167.1 min, and 1% buffer B from 167.1 to 180 min) at a flow rate of 400 nL min^−1^ and a spray voltage of 1.5 to 1.8 kV. Finally, 2% buffer B was used to re-equilibrate the column in 15 min using an oven set to 55 °C and a heated desolvation capillary set to 275 °C. Xcalibur (Rev.2.1.0) was used to operate the LTQ Orbitrap Elite mass spectrometer via its instrument method files in the positive-ion mode. Linear ion trap and Orbitrap instruments were operated in parallel in which a full Orbitrap MS scan detected tandem MS (MS/MS) spectra of the ten most intense precursors, from the most to least intense, in the range of 150 to 2,000 m/z at a resolution of 60,000. The relative collision energy for rapid collision-induced dissociation (rCID) was set to 35% using dynamic exclusion with a repeat count of 1 and a 45-s exclusion duration window. Singly and unknown charged ions were rejected for MS/MS and the corresponding mass spectra recorded with Xcalibur software 2.2 SP1.48 (Thermo Scientific).

The Andromeda software^32^ was used to identify proteins from mineral and continuous cultures, and MaxQuant 1.5.3.175^31^ label-free protein quantifications (LFQ) algorithm^33^ used for quantification. The genomes of the three bacteria (see above) were used for protein identification based on the corresponding FASTA files. After quantification, the Perseus software v1.5.8.5^34^ was used to discard rows with <2 values of either condition (mineral or continuous), and to compare the intensities with two-sample Welch’s *t* test.

### Microscopy imaging

#### Mineral sample preparation for microscopy imaging

Chalcopyrite flotation concentrate obtained from Boliden AB, Sweden was wet sieved (Retsch, Germany) to obtain the 50–100 *μ*m particle size fraction. The resulting mineral grains were washed twice for 30 min in 10 volumes of a solution of 0.1 M EDTA and 0.4 M NaOH, stirred twice in approximately five volumes of acetone for 30 min to remove soluble sulfur compounds^13^, and finally sealed and heated in a nitrogen atmosphere for 10 h at 125 °C.

#### Microbial species cultivation for microscopy imaging

Epifluorescence microscopy (EFM) pictures were taken of biofilms formed by the three bacterial strains used for transcriptomics and proteomics. *A*. *caldus*, *S*. *thermosulfidooxidans*, and *L*. *ferriphilum* were cultured in sterile MAC medium with soluble electron donors for inoculation of chalcopyrite cultures as described below. For *L*. *ferriphilum*, 4 g/L iron(II)-ions were provided as FeSO4 · 7H_2_O and the pH was adjusted to pH 1.6–1.8 with H_2_SO_4_ to prevent precipitation of the electron donor. *A*. *caldus* and *S*. *thermosulfidooxidans* were pre-cultured using 0.9 g/L potassium tetrathionate (K_2_S_4_O_6_) with the further addition of 0.02% yeast extract (YE) and 0.1 g/L iron(II)-ions for *S. thermosulfidooxidans*^11^. Cultivation proceeded until reaching stationary growth phase after 4–5 days of incubation. Cells were harvested by centrifugation at 11,270 g for 10 min, washed in sterile medium, and used to inoculate chalcopyrite-containing Erlenmeyer flasks (150 mL MAC medium and 2% (wt/vol) chalcopyrite grains of size 50–100 *μ*m) at an initial cell density of 10^7^ cells/mL (in equal proportions for mixed cultures). Mineral cultures were grown for a different time span of 1, 3, 7, 14, and 21 days after inoculation.

#### Microscopy sample preparation

Fixation of mineral-attached cells was achieved by transferring about 25 mg of mineral material from the cultures to 1 mL sterile MAC medium at pH 1.8 with 4% formaldehyde and incubation at room temperature for 1 h. The samples were then washed twice with water followed by a single wash with 1 mL phosphate-buffered saline (PBS) and frozen at −20 °C in 50% ethanol in PBS until ready to be examined. For visualization, the mineral particles were incubated in 200 *μ*L 0.01% 4′,6-diamidine-2′-phenylindole dihydrochloride in 2% formaldehyde. The mineral grains were then washed with 1 mL PBS before and after staining mineral-attached cells, and mounted on 10-well diagnostic glass slides (10-well, 6.7 mm; Thermo Scientific) using a glycerol-based mounting medium (CitiFluor AF2) and covered with glass coverslips^14^. Observations of biofilms before and after fixation did not show any disruptive effects of the fixation.

#### High-throughput epifluorescence microscopy

**Fig. 2:**
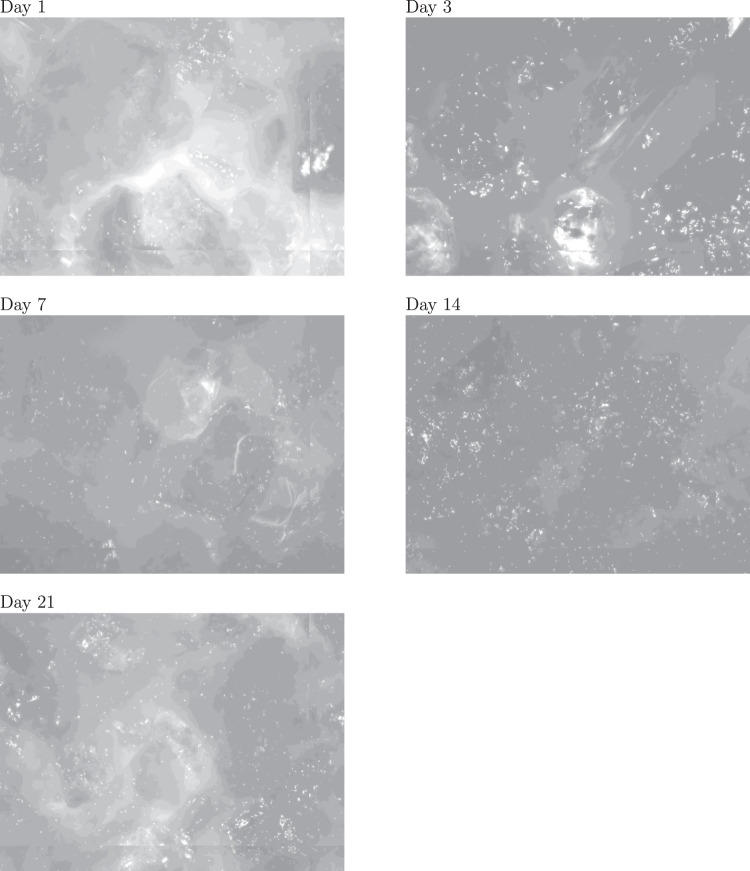
Example of EFM images of a mixed microbial community composed of *L*. *ferriphilum*, *S*. *thermosulfidooxidans*, and *A*. *caldus* taken at different time points.

An EFM platform AxioImager M2m (Zeiss) with a motorized microscopy stage (IM SCAN 130 × 85 - DC 1 mm, Märzhäuser Wetzlar) and an AxioCam MRm camera was used to automate image acquisition in order to generate sets of images for different acidophile microbial cultures. The individual images of stained microbes on the mineral grains were recorded using a Zeiss Plan-Neofluar (20 × 0.50) objective and corresponded to an imaged area of 450 × 335 *μ*m. The obtained images were taken with a 2-*μ*m step size and stacked to cover the entire maximum grain depth of 100 *μ*m (*i*.*e*., a total of 50 layers). The extended-focus module of the Zen 2 software (blue edition, 2011; Carl Zeiss GmbH) was used to calculate projection images using the Wavelet option and the resulting projections were exported as JPEG files. For each mineral sample and time point, quantification of the cells on the mineral surfaces used a minimum of 36 images. Images were taken at days 1, 3, 7, 14, and 21 (Fig. 2).

## Data Records

**Fig. 3:**
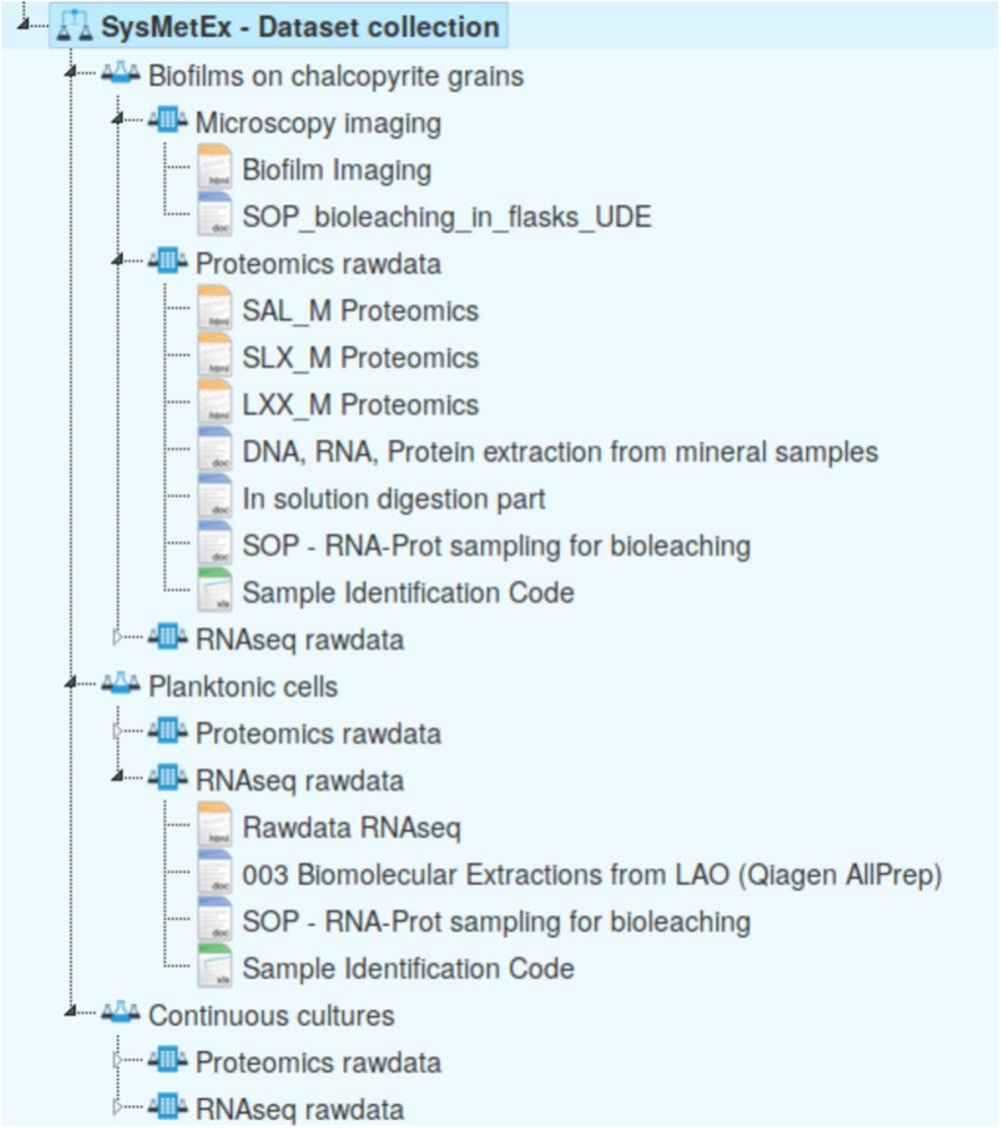
Overview of the FAIRDOMHub raw data repository. Raw data files are accessible for each cellular fraction, *i*.*e*., biofilm on mineral particles, planktonic cells from leaching experiments, and continuous culture samples alongside standard operating procedures applied when processing the tables. Links to the raw data repositories for the respective data types are provided.

The raw reads of the RNAseq data for 12 continuous culture samples and 49 bioleaching samples were deposited at ENA^35–40^. Proteomics data for three continuous culture samples and 61 bioleaching samples was deposited at the PRIDE database^41–53^. A per-sample overview of the available omics data with the respective accessions can be found in table “omics samples” at the FAIRDOMHub^54^ repository “SysMetEx – Dataset collection”^55^, in which raw omics data are summarized in a structured format (Fig. 3). Sample identifiers provide information, such as laboratory of origin, inoculated strains, and inoculum size, cellular fraction, as well as run time of the cultures. Additional information to decode the contained data is also provided in the same repository. The number of samples for which RNAseq or proteomics data was generated is summarized in Table 1.

## Technical Validation

Cell pellets from the bioleaching experiments were sampled aseptically and changes in nucleic acid and protein immediately inhibited by rapid cooling followed by flash freezing in liquid nitrogen (as described in the methods). All samples were shipped on dry ice to ensure the samples remained frozen. Biomolecular extractions were performed according to standard procedures following best practices to avoid RNA degradation and contamination. Quality and concentration of extracted biomolecules were checked before proceeding to library preparation for RNAseq or proteomics analyses (see methods).

**Fig. 4:**
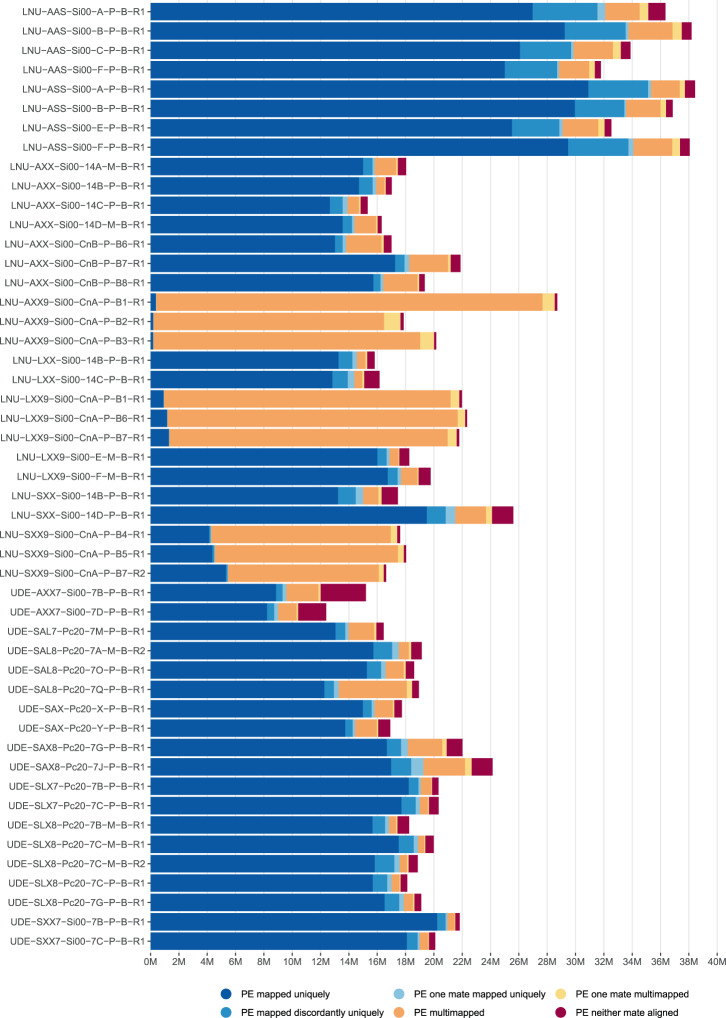
Summary of mapping files. Preprocessed RNAseq reads were mapped to a concatenation of the three reference genomes. The resulting alignments were summarized and classified with samtools flagstat indicated by bar color. The plot is an excerpt of the multiqc report for the mapping files and featurecount results (FAIRDOMHub repository “SysMetEx – Dataset collection”^55^, file “mapping statistics”). A description corresponding to the sample names on the left is found in the FAIRDOMHub repository (“SysMetEx – Dataset collection”^55^, “Sample Identification Code”).

Quality of sequencing reads for the RNAseq data was assessed with FastQC and summarized across all samples with multiQC^56^. Some samples failed the FastQC check for quality scores. However, after preprocessing, all reads showed high quality scores (FAIRDOMHub repository “SysMetEx – Dataset collection”^55^, files “qc raw reads” and “qc processed reads”) and successful adapter removal. Overall read duplication levels might appear excessively elevated. However, this is likely due to incomplete rRNA removal. Especially for the continuous culture samples, for which no rRNA removal was performed and duplicate read counts constitute a large portion of the total raw reads. This is also reflected in reads mapping to reference genomes. These samples show an elevated number of multi-mapping reads in contrast to depleted bioleaching samples (Fig. 4). Overall, the amount of reads mapping to the reference genomes provides sufficient depth for detailed analyses with 7 to 35 million reads assigned to the reference protein coding genes in bioleaching samples (FAIRDOMHub repository “SysMetEx – Dataset collection”^55^, file “mapping statistics”).

**Fig. 5:**
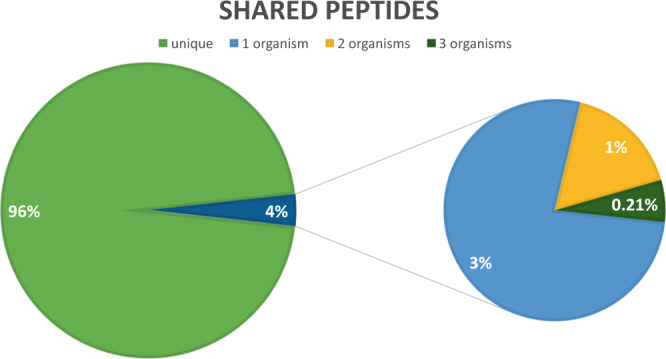
Overview of identified peptide sequences shared between two and more proteins for a cultivation condition comprising all three organisms. The 4% shared peptides were further broken down into peptides shared by proteins from 1, 2, or 3 organisms.

Quality of proteome data was assessed using MaxQuant output. First, the identification rate of MS/MS spectra was surveyed (Table 2). The identification rates varied between conditions, without clear bias towards any of them. The average identification rate was 22%, which is in the typical range for high-throughput proteomics using CID as fragmentation technique. As in shotgun proteomics, peptide sequences are used to infer protein presence and abundance and a common problem is shared peptide sequences – here, not only in one organism but up to three organisms per sample. To gauge the magnitude of shared peptides and their detrimental effect, an example proteome from a cultivation comprising all three organisms was analyzed. It can be seen (Fig. 5) that shared peptides did not severely impair protein inference. Altogether 96% of all peptides were unique. Interestingly, of the 4% shared peptides, 3% belonged to the same organism. Only about 1% and 0.2% were shared between 2 and 3 organisms, respectively. Therefore, in this study the individual proteomes of the used three organisms were dissimilar enough to avoid potential complications of inter species-shared peptides.

In this project we used a motorized EFM for automated image acquisition coupled to automated image analysis using algorithms that allowed quantification of mineral-attached cells. The evaluation of the method’s statistical accuracy depended on the number of images considered. After manual removal of extreme values, representing the top and bottom deciles of images with extremely low or high cell counts, metal sulfide colonization values (cells per mm^2^) of at least 36 images were used. Then, the values were randomly sorted using Microsoft Excel’s random function and grouped in four arbitrarily chosen classes with data from nine microscopy images in order to calculate the mean of each class. These four classes can be understood as four sets of equal mineral areas used for averaging of the naturally non-homogeneous mineral colonization over a larger area than that represented in a single microscopy image. The coefficient of variation was found to not exceed 16 ± 8% when at least 36 images per sample were analyzed. In order to take into account the fact that the mineral grains were viewed only from the top, the resulting values were doubled in order to account for the unobserved bottom side, while no correction factor was used for extrapolation from two-dimensional areas to the true three-dimensional mineral objects.

## Data Availability

The workflow utilized for quality filtering the RNAseq reads, alignment to references genomes, and counting of mapped reads has previously been described^11^, and can be found in the “SysMetEx – Data analysis” repository^57^. Reference genomes of the three strains used in the project and auxiliary files can be accessed at the “SysMetEx – Reference genomes” repository^58^. Proteomics data processing was carried out according to the MaxQuant parameter (FAIRDOMHub repository “SysMetEx – Dataset collection”^55^, file “maxquant parameters”).
